# The Influence of Study Selection Criteria on Statistical Conclusion Validity: A Simulation Study

**DOI:** 10.1007/s40688-026-00580-z

**Published:** 2026-03-21

**Authors:** Allyson L. Hayward, Jeffrey A. Shero, Jessica A. R. Logan

**Affiliations:** Department of Special Education, Vanderbilt University, Nashville, USA

**Keywords:** Tier 2 reading interventions, Struggling readers, Simulation study

## Abstract

Randomized controlled trials (RCTs) are the gold standard for evaluating intervention effectiveness. In education research, students are often screened to identify those with reading difficulties (SWRD) who are eligible to participate. However, criteria for identifying SWRD vary widely across studies and are often shaped by practical constraints such as budget or statistical power. Common approaches include using publisher-defined risk categories, percentile-based thresholds, or selecting a fixed number of the lowest-performing students per classroom. These study-specific decisions, though necessary, may introduce unintended bias. In this simulation study, we examined how different eligibility criteria, applied to the same underlying population with a fixed “true” effect, can influence estimated intervention effects. Results showed that all tested methods produced some degree of bias in effect size estimation, but the magnitude and direction of bias were inconsistent and unpredictable. These findings suggest that variation in how researchers define SWRD can distort the field’s understanding of intervention efficacy. We suggest future areas of research to guide the field toward a solution that is both statistically valid and practically feasible.

## Introduction

The randomized control trial (RCT) is generally well accepted as the gold standard for testing the efficacy or effectiveness of new programs or interventions (Cook & Cook 2013; Slavin, 2019). In the context of education research, the results of high quality RCTs are commonly used to select practices and programs for adoption by education agencies; ensuring that inferences from RCTs are high quality is a major activity of education research clearinghouses such as the What Works Clearinghouse (WWC, 2020) and National Center on Intensive Intervention (NCII). The RCT maximizes internal validity, prioritizing the comparison between the treatment and counterfactual conditions, but does so often at the expense of other aspects of validity (Shadish et al., 2002; Cronbach et al., 1982). In the present study, we examined whether and to what extent one aspect of the RCT, the determination of who is eligible for intervention, is associated with statistical conclusion validity of the estimates of the treatment effect.

It is a feature of the RCT that only some participants would be eligible or in need of intervention, and it is only those participants who should be included in the sample and the randomization. Eligibility criteria can be understood as study-specific choices involving decisions that govern who will be screened in or excluded from a sample. This is especially relevant for Tier 2 interventions, which are widely implemented within the response to intervention (RTI) framework. RTI is a multi-tiered system of support that provides increasing levels of instructional intensity based on students’ demonstrated needs. Tier 2 interventions consist of targeted small-group instruction for students who are not making adequate progress in Tier 1 (core classroom) instruction and who require additional, focused support (Fuchs & Fuchs, 2006). Therefore, by definition, Tier 2 interventions require students to be identified for further support using eligibility criteria.

Currently, little is known about how eligibility criteria affect the validity of research findings. Limited research has been conducted to investigate the implications of selective eligibility criteria on statistical conclusion validity. However, in a medical study, Weisberg and colleagues (2009) exemplified how changes in eligibility criteria for treatments bias research findings. They used a counterfactual framework to model the bias in the estimation of effects of an antidepressant medication when using different eligibility criteria. They observed that as criteria become stricter (i.e., displaying severe symptoms of depression), bias in effect estimation becomes more extreme. This work demonstrates the potential for eligibility criteria to affect estimates of the effectiveness of interventions. However, their work only included members of the target population, and screening was to determine who would have a low risk to side effects. They did not test criteria to identify who is and is not a part of the target population itself.

### Eligibility Criteria and Reading Interventions

Investigating the potential impact of eligibility criteria is particularly relevant to efficacy studies on Tier 2 reading interventions. These provide supplemental, small group instruction to students who do not show adequate skill acquisition from Tier 1 instruction alone. The purpose is to advance reading growth so that students may catch up to perform on par to grade-level expectations. However, showing adequate skill acquisition is not an operational definition. It can be interpreted differently relative to which or how many skills are lacking, or the severity of the inadequacy.

The present study is set within the context of special education, where eligibility criteria are set to identify members of a population and to exclude those who do not qualify. Related to this, Sofie and Riccio (2002) compared different screening measures to assess if they were comparable for identifying individuals for having a reading disability. They found groups derived from different screeners to be significantly different from each other, particularly between the use of standardized measures of reading achievement, phonological processing measures, and curriculum-based measures of fluency. While their work demonstrated that different measures can and do identify different individuals as being eligible for intervention, it did not examine how those differences affected the estimates of intervention effectiveness.

#### Common Eligibility Criteria for Struggling Readers

Identification of reading disability is complex. Definitions of this construct do not provide procedures for diagnosis (Wagner and Lonigan, 2023). Thus, there is no consensus on selection criteria across studies for any reading disability. This is an issue to which every efficacy study generalizing to any population of students with some reading disability contributes. Two studies may both define their samples as struggling readers but have acquired their samples based on different criteria. Though these inconsistencies are common, not much is known about whether or to what extent that is an issue. There is very little research investigating if this is problematic for the validity of the evidence base for effective reading interventions.

Given the potential importance of eligibility criteria to the field, we first wanted to examine whether and the extent to which research teams employ different eligibility criteria in their studies. To do so, we turned to a meta-analysis of Tier 2 reading interventions conducted by Wanzek and colleagues (2016). For each of the 72 studies, we coded how the researchers determined whether a student was eligible for Tier 2 instruction. We broadly categorized the observed strategies into three groups. Eight studies screened in those who met the criteria of some risk range established by test developers (e.g., 40th population percentile). Twenty-one studies used a cutpoint corresponding to a given percentile of all screened students in the sample (e.g., 20th screening sample percentile). Lastly, twenty-eight studies selected some number of the lowest scoring students per classroom or school (e.g., the lowest 4 students per class). Six studies did not specify their eligibility criteria. The remaining nine studies could not be categorized (e.g., had to qualify for summer school; had to have an IEP with reading goals). This raises the question of whether these methods of sample acquisition are equivalent.

The present study investigates the comparability of these common selection methods using simulation approaches. Simulation is a powerful tool in this investigation for several reasons. First, variables may be manipulated and samples can be drawn in ways that are not ethically or logistically possible with real participants. Second, with simulation we can isolate observed differences between conditions to a single source. In this case, any differences in the outcome will solely be due to the eligibility criteria used because the simulation controls all other factors to remain the same. We used Monte-Carlo simulation to examine if eligibility criteria alone is a source of variance in estimates of intervention effect sizes. Monte-Carlo style simulation allows for explorations of the stability and consistency of eligibility criteria’s impact on intervention effect sizes, replicating the simulations many times over to allow for natural variations to occur. To understand potential consequences of selection criteria for statistical conclusion validity, we ask: Do different methods of eligibility criteria yield differential estimates of the treatment effect for large samples?

## Method

### Simulating the Data

To simulate the data, we first turned to how effect sizes of reading interventions have been tested in the published literature. The reviewed intervention studies in Wanzek (2016) most commonly used a statistical model that examined the relation between students’ assigned intervention condition (treatment or control) and their posttest outcomes while controlling for pretest scores and other covariates such as student characteristics.

To follow this practice, we simulated a data set to represent the constructs of age, classroom, assigned condition, pretest, and posttest for 5,000 observations (referred to from now on as students). Using the MASS package (Venables & Ripley, 2002) in R (Version 4.2.1), normally distributed data was simulated using a specified covariance matrix between the variables age, pretest, and posttest. To align with correlations typically observed in the field, we simulated data where pretest and posttest were correlated at 0.6, and both pretest and posttest were correlated with age at 0.2. All variables were represented as z-scores (*M* = 0, *SD* = 1).

Once the variables specified in the covariance matrix were simulated, classroom identification and condition assignment variables were appended. The classroom identification specified each student to one of 250 classrooms with exactly twenty students per classroom. The dataset assumes no intraclass correlation. The condition assignment randomly assigned half of the sample (*N* = 2,500) to the control condition and the other half to the intervention condition. Next, posttest scores were adjusted based on condition assignment; assignment was conducted at the child-level. Next, the treatment effect was simulated as an average effect size of *d =* 0.3, slightly smaller than the average effect size found by Wanzek et al. (2016) on standardized language and comprehension measures (Hedge’s g = 0.36). To simulate the treatment effect, a column was created of random normally distributed values centered around the assumed effect size estimate (*M* = 0.3, *SD* = 1). For each student who was assigned to the intervention condition, their posttest score was adjusted by adding their corresponding effect estimate to their originally simulated posttest. Students in the control condition did not have their posttest adjusted.

Code for the simulation, the data simulated, and subsequent analyses are available on the Open Science Framework (https://osf.io/dyahp/overview?view_only=5ec2b0761d0a4ac5bb447938cc2bd5e4*).*

### Sample Selection and Statistical Power

Next, three samples were selected from the 5,000 students based on how eligibility criteria were determined within the studies included in systematic review conducted by Wanzek and colleagues (2016) on tier two reading interventions. The three eligibility criteria selected different students for inclusion depending on their pretest scores. Method 1 selects students at or below the 40th percentile, and was designed to be reflective of selecting students who failed a population-based screener. Method 2 selects students at or below the 25th percentile, and was designed to represent the selection of students falling at the lowest percentile of students who were screened. Method 3 selects the lowest scoring 4 students per classroom. Each of these methods were designed to reflect common eligibility criteria used in practice. In principle, Methods 1 and 2 differ in that one applies a population-based cutoff and the other a sample-based cutoff. However, because the simulated data were standard scores (*M* = 0, *SD* = 1), the population and sample distributions were functionally equivalent. Thus, Methods 1 and 2 were implemented in the same way, differing only in their percentile thresholds. Each sampling method yielded a new variable indicating for each student if they were deemed eligible for the study.

Prior to conducting this simulation, we first wanted to determine statistical power for the proposed analyses. This would ensure that any differences between conditions observed were not due to differences in statistical power. Because data are normally distributed and the number of students in the full data set is known, each sample size can be approximated to calculate power. Method 1 would identify all observations 0.25 standard deviations below the mean to be eligible, which would equate to 40% of the full dataset. This would identify approximately 2000 students as eligible. Method 2 would identify all students below the 25th percentile as eligible, and would yield approximately 1250 students as eligible. Lastly, method 3 would identify four students in each of 250 classrooms, and would result in a sample of 1000 students. For the primary research question of the effectiveness of the hypothetical intervention (group difference effect size set as *d* = 0.30), we conducted an exploratory power analysis for power of 0.80 and an alpha level of 0.05. Results indicated that power does not continue to increase substantially beyond sample sizes of 600. Using the approximated sample sizes per method, we determined that statistical power was not a concern for the primary estimate of intervention effectiveness within each drawn sample.

### Analysis

In line with how many intervention efficacy studies evaluate the impact of a treatment, we analyzed each dataset using multiple linear regression. Posttest scores were regressed onto intervention assignment, age, and pretest within each sample. This model was run four times, one for each subsample of the simulated data yielded from each of the three selection criteria as well as with the full dataset. We extracted information from the results of each model to compare. Specifically, we extracted the intervention effect size (estimated as the parameter estimate of the intervention condition variable) and its standard error, the percentage of bias on said estimate (measured as the difference between the observed effect estimate and the observed effect estimate from the full sample), and finally the overall variance explained in each model. Regarding the intervention effect size, this parameter was considered an estimate of the effect when taken from each of the models using subsamples of eligible students. It was considered the true effect when extracted from the model that used the full (screening) sample. It is worth noting that this choice may result in a slight over-estimation of the true effect.

### Monte-Carlo Approach

A Monte-Carlo simulation approach was taken whereby the simulation and subsequent modeling processes were replicated 500 times and allowing for natural variation to occur. In each iteration, data were simulated based on the same covariance matrix and sample size, samples were selected with the same criteria, and the same regression models with the same covariates were run. The only difference between iterations was due to randomness during simulation, which by design is intended to represent the natural variation that exists within and across real world data. Results are presented first as measures of central tendency and second as measures of dispersion across all replications. First, the means of the estimates and the means of their corresponding standard errors are reported. Then, the ranges and standard deviations of the same estimates are reported. Reporting the ranges and standard deviations of these results allows us to observe how predictable the impacts of selection criteria are, and if the resulting biases due to selection criteria are consistent in magnitude and/or direction. Like Simulation 1, the estimates reported include the estimated intervention effect size and its standard error, the percent bias, and the overall variance explained by each model.

## Results

### Distribution

**Fig. 1:**
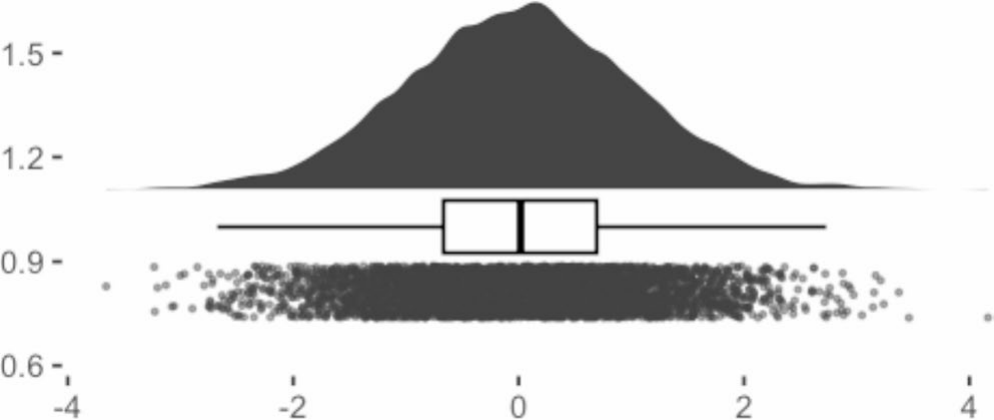
Raincloud plot of pretest scores of full simulated sample

**Fig. 2:**
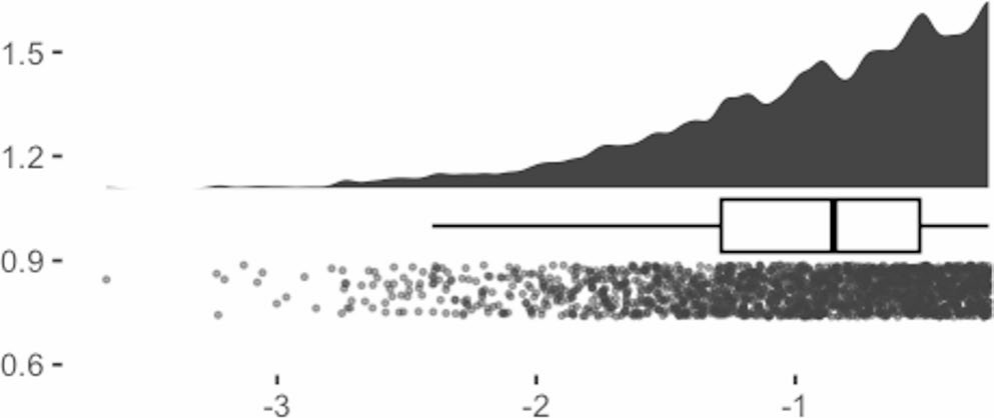
Raincloud plot of pretest scores of method 1 eligible sample: 40th percentile

**Fig. 3:**
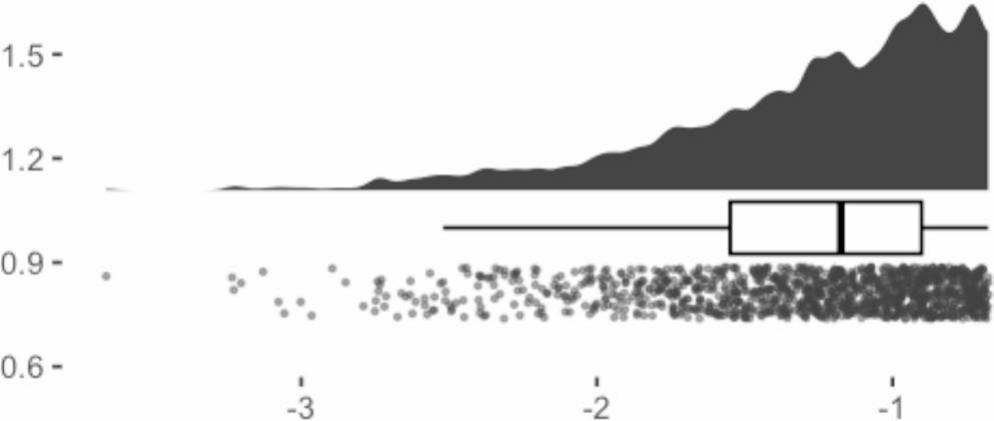
Raincloud plot of pretest scores of method 2 eligible sample: 25th percentile

**Fig. 4:**
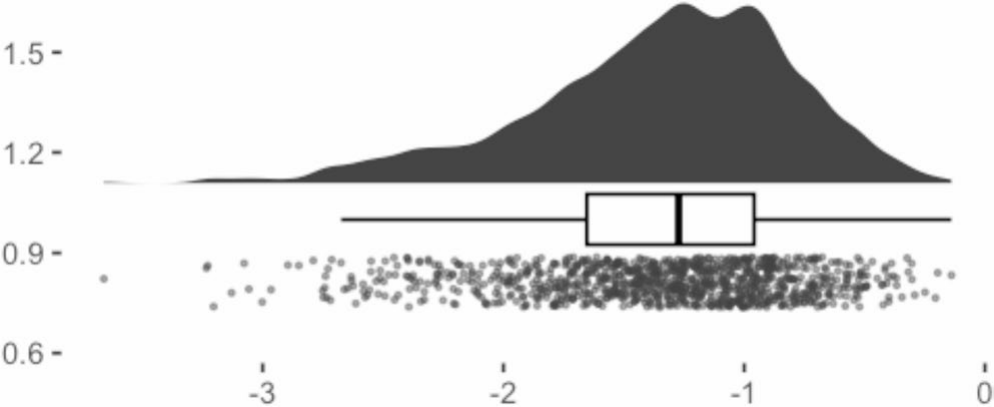
Raincloud plot of pretest scores of method 3 eligible sample: lowest 4 per classroom

To visualize the distributions of the selected samples, we created raincloud plots from a single iteration of the data simulation (see Figs. 1, 2, 3 and 4). Given that the screening sample (full dataset) was normally distributed, we can see how the application of different criteria alter the shape of the eligible samples. Methods 1 (40th percentile) and 2 (25th percentile) yielded very negatively skewed samples (*skewness* = −1.01 and − 1.12, respectively). Method 3 (four lowest children) while still negative, had a less extreme skewness of − 0.62.

### Regression Modeling

Mean model results across the 500 replications are presented in Table 1. The mean estimate for the intervention effect was 0.30 for the full sample as well as all three selection methods. On average, there was no bias, meaning that each method recovered the known true effect after 500 replications. The standard error of that estimate was small for the full sample (0.03) and roughly double that for Methods 1, 2, and 3 (0.05, 0.06, and 0.07, respectively). The average amount of variance explained was consistently smaller for all selection methods relative to the full sample. For the full sample, 26% was explained while 11%, 9%, and 11% was explained for Methods 1, 2, and 3, respectively.

**Table 1:** Mean model results across replications

	Full Sample	Method 1: 40th percentile	Method 2: 25th percentile	Method 3: Lowest 4
Intervention (SE)	0.30 (0.03)	0.30 (0.05)	0.30 (0.06)	0.30 (0.07)
% Bias	-	0%	0%	0%
*r*-squared	0.26	0.11	0.09	0.11

Note. Percent bias was calculated as the. true effect minus the observed effect divided by the true effect; *SE =standard error and represents average standard error associated with the given parameter across the 500 replications*

Ranges across iterations of the results were also calculated and can be seen in Table 2. The true effect ranged between 0.20 and 0.39. The percent bias associated with each true effect covered a large range. Each method was roughly balanced in the extent of its over- and underestimation, explaining the average percent bias of 0% observed but large range of overall observed biases. Method 1 underestimated as low as 41% and overestimated as high as 46%, Method 2 as low as 58% and as high as 61%, and Method 3 as low as 60% and as high as 49%. This indicates that although the average bias across iterations was 0%, the potential bias for any single iteration applying these approaches was considerably higher. The full sample had a standard deviation of 0.03 while Methods 1 (0.05), 2 (0.06), and 3 (0.07) were roughly twice that. These results suggest that, on average, each method adequately recovers the true effect. However, the distribution of those results exhibits that there is considerable variance around the mean, indicating that any single application of these selection criteria may have a wide and unpredictable range of potential biases, both in magnitude and direction.

**Table 2:** Ranges of model results (Minimum to Maximum) across replications

	Full Sample	Method 1: 40th percentile	Method 2: 25th percentile	Method 3: Lowest 4
Intervention (SD)	0.20–0.39 (0.03)	0.14–0.46 (0.05)	0.11–0.48 (0.06)	0.10–0.50 (0.07)
% Bias (SD)	-	−42% – 46% (12%)	−58% – 61% (18%)	−60% – 49% (20%)
*r*-squared	0.22–0.28 (0.02)	0.07–0.16 (0.02)	0.05–0.15 (0.02)	0.06–0.17 (0.02)

Note. Percent bias was calculated as the true effect minus the observed effect divided by the true effect; SD = standard deviation and quantifies the dispersion of the effect estimate across the 500 replications of the simulation

## Discussion

On average, each selection method produced unbiased estimates of the true effect, indicating good recovery of the underlying parameter. However, measures of dispersion told a different story: the standard errors from the selected subsamples were roughly twice as large as those in the full screening dataset. In other words, although the true effect was recovered on average, there was considerable uncertainty around any single estimate. This distinction is critical because, in practice, researchers apply selection criteria once, not repeatedly across many replications. While the bias introduced by selection criteria may average out to zero across studies in a field, the results of any individual study are likely to be substantially biased in an unpredictable direction.

The measures of dispersion provided establish the instability of results yielded from each selection method. The bias not only had a large range, but that range covered both positive and negative values. This means that although the average bias is 0%, this is simply a result of the bias not occurring in a systematic way, such as if it were to consistently increase the uncovered effect sizes, but rather was random and unpredictable in nature in the way it would impact said findings. As such, at any given iteration for each selection method, the intervention effect could have been inaccurate in either direction of under- or overestimating the effect. The instability of the bias highlights the fact that error due to selection criteria is not something researchers would be able to adjust or control for in their models.

Among the approaches evaluated, Method 1 appeared to yield the most acceptable performance. This may have been because it retained a larger sample size or was less affected by restriction of range (as evidenced in Fig. 1), both of which likely contributed to more stable estimates. Nonetheless, even this method did not fully eliminate threats to statistical conclusion validity. Using Method 1 may therefore represent a practical, if imperfect, approach for researchers to employ in the interim, while further work is conducted to more comprehensively address the sources of bias introduced by selection criteria. Together, these findings highlight that while unbiased recovery of the true effect can occur on average, substantial variability across individual applications poses a persistent concern for the reliability of study-level conclusions.

### Implications for the Field

The eligibility criteria operationalized in our study were selected to represent those commonly used in Tier 2 reading intervention research. By modeling these typical approaches to sample selection, we demonstrated that methods for identifying struggling readers can introduce substantial uncertainty into estimates of intervention effectiveness. In doing so, our findings suggest that the selection criteria frequently employed in Tier 2 intervention studies may compromise the statistical conclusion validity of their results. Below, we discuss three key ways this issue manifests in the field and shapes our understanding of what constitutes an effective Tier 2 intervention.

First, the additional variability in results provided by eligibility criteria should be taken into consideration when evaluating and reviewing previous literature. When evaluating the evidence base for Tier 2 reading interventions, it should be kept in mind that findings of effectiveness all have some degree of bias simply due to how they selected their sample. These implications could also be considered when conducting meta-analyses. For example, A *u* ratio can be used to quantify and correct for the impact of selection effects (Wiernik & Dahlke, 2020). This is a ratio between the standard deviation of a variable in the population and that of the specific sample. Examining potential corrections for the uncertainty introduced due to selection criteria using indices such as the *u* ratio is a direction for future research.

Second, when planning future work, researchers should be aware of the bias introduced to their models from the eligibility criteria they operationalize. By design, Tier 2 interventions are meant for students who are on the lower end of the distribution of achievement. Given that eligibility criteria is an essential component of Tier 2 studies, we must understand this as an inherent limitation to the statistical conclusion validity of findings. Ideally, future work will seek to make corrections for this type of bias. Though, given its unpredictable nature a statistical correction may not be possible.

Third, when pretest scores are used as eligibility criteria, the variance of that covariate is inherently truncated, reducing the amount of variability it can explain in subsequent analyses. Because Tier 2 interventions are designed for students performing below benchmark, this commonly included covariate will, by definition, violate assumptions of normality. This restriction of range not only distorts the estimated effect size but also diminishes overall model fit and explanatory power. As Cohen et al. (2003) note, when one variable has a restricted range, “the proportion of variance in Y associated with X will necessarily be smaller, and therefore r-squared will be smaller” (p. 57). Based on the assumptions of power analyses, models in this line of research are by design set up to overestimate their power to detect differences.

From a practical standpoint, the results of this study highlight the importance of the eligibility criteria used and how decisions regarding this will bias results. Despite this, however, we must acknowledge that selection criteria is essential to Tier 2 intervention studies. We found that there is no tested method that is the smallest threat to statistical conclusion validity. Rather, all tested methods contributed considerable and unpredictable bias to the estimation of intervention effect. We understand that there is a practical need for selection criteria and there does not appear to be a method that is immune to bias. Because of this our study does not intend to suggest the best eligibility criteria to use for tier 2 interventions. Rather, it highlights bias associated with several of the most common criteria used in our field to call for more research to understand how we may overcome this issue.

### Alignment with Other Research

Weisberg et al. (2009) also found that observed effects can be impacted by selection criteria. However, their work focused on selection bias rather than identifying true members of a target population. They assumed all screened individuals to already be a part of their target population while we assume we are trying to screen out individuals who would not be considered a part of that population. Sofie and Riccio (2002) was more conceptually aligned to our study and our results compliment each other. While we both sought to investigate differences in samples based on eligibility criteria, we examined different kinds of outcomes. They observed significant between-group differences on reading measures while we observed differences in intervention effectiveness. Together, there is evidence to suggest that selection criteria are not always comparable to each other and have trickling effects on who evidence can generalize to and the extent of its validity.

### Limitations

The limitations of our study center around the strict assumptions from which the data were simulated. First, the data for our study was perfectly multivariate normal and there was no variance attributable to nesting. Data that so perfectly adheres to such assumptions is not common. It is likely to observe some degree of skewness or kurtosis in distributions, especially when trying to screen students who tend to perform low. Moreover, an ICC of zero is unlikely in applied educational data, where some variance is always attributable to classroom membership. Second, to simulate an intervention effect, posttest scores were created based on a consistent effect size and the assumption that the intervention is equally effective, on average, for anyone who received it. However, it may be the case that interventions designed specifically for students at risk for learning disabilities are only effective for those students, and instead have little to no impact on their average to high performing peers. Under these conditions, selection criteria may have a more pronounced effect, something we intend to examine in a follow up study. Given that interventions are often designed with this population in mind, it is likely more common to see interventions be effective more for some students and less for others. Taken together, the limitations suggest our data meets assumptions that are not common to observe in data from real children in real classrooms.

### Future Directions

Our work suggests several important directions for future research, with particular emphasis on examining moderated treatment effects. In the current study, we simulated data assuming a uniform intervention effect across all students. However, in practice, interventions often yield compensatory effects, whereby students who begin with lower skill levels demonstrate greater gains. Future research should therefore investigate how different forms of moderation by pretest performance influence bias in parameter estimates and the accuracy of selection criteria. Additionally, research could explore how bias patterns vary under different study conditions. For instance, smaller screening sample sizes are known to reduce statistical power and increase standard errors (VanVoorhis & Morgan, 2007), potentially compounding the influence of selection procedures. Likewise, varying data structures, such as differing levels of intraclass correlations (ICCs) or the inclusion of fixed versus random intercepts and slopes, may amplify or attenuate estimation bias. These extensions would clarify how methodological and contextual factors jointly affect study validity.

Finally, we call for future research that not only identifies sources of bias but also develops and evaluates potential solutions. Advancing the field will require practical and statistically rigorous approaches for testing Tier 2 intervention efficacy, followed by empirical evaluations of their robustness across diverse data and design conditions.

## Conclusion

Evidence of effective interventions is dependent on the samples from which the data are produced. Across studies that seek to generalize to the same population, samples are selected inconsistently. The results of our study would suggest that these inconsistencies impact statistical conclusion validity, and some selection methods are more biased than others. Further investigation is required to understand the extent of that claim under less strict assumptions.
